# Antioxidant Serine-(NSAID) Hybrids with Anti-Inflammatory and Hypolipidemic Potency

**DOI:** 10.3390/molecules26134060

**Published:** 2021-07-02

**Authors:** Panagiotis Theodosis-Nobelos, Georgios Papagiouvannis, Paraskevi Tziona, Panos N. Kourounakis, Eleni A. Rekka

**Affiliations:** 1 Department of Pharmacy, School of Health Sciences, Frederick University, Nicosia 1036, Cyprus; hsc.pag@frederick.ac.cy (G.P.); panoskur@pharm.auth.gr (P.N.K.); 2 Department of Pharmaceutical Chemistry, School of Pharmacy, Aristotelian University of Thessaloniki, 54124 Thessaloniki, Greece; tzionap@gmail.com (P.T.); rekka@pharm.auth.gr (E.A.R.)

**Keywords:** antioxidant serine conjugates, inflammation, lipoxygenase inhibition, dyslipidemia, antioxidant activity, anti-inflammatory agents

## Abstract

A series of L-serine amides of antioxidant acids, such as Trolox, (*E*)-3-(3,5-di-*tert*-butyl-4-hydroxyphenyl)acrylic acid (phenolic derivative of cinnamic acid) and 3,5-di-*tert*-butyl-4-hydroxybenzoic acid (structurally similar to butylated hydroxytoluene), was synthesized. The hydroxy group of serine was esterified with two classical NSAIDs, ibuprofen and ketoprofen. The Trolox derivatives with ibuprofen (7) and ketoprofen (10) were the most potent inhibitors of lipid peroxidation (IC_50_ 3.4 μΜ and 2.8 μΜ), several times more potent than the reference Trolox (IC_50_ 25 μΜ). Most of the compounds decreased carrageenan-induced rat paw edema (37–67% at 150 μmol/kg). They were moderate inhibitors of soybean lipoxygenase, with the exception of ibuprofen derivative 8 (IC_50_ 13 μΜ). The most active anti-inflammatory compounds exhibited a significant decrease in lipidemic indices in the plasma of Triton-induced hyperlipidemic rats, e.g., the most active compound 9 decreased triglycerides, total cholesterol and low-density lipoprotein cholesterol by 52%, 61% and 70%, respectively, at 150 μmol/kg (i.p.), similar to that of simvastatin, a well-known hypocholesterolemic drug. Since the designed compounds seem to exhibit multiple pharmacological actions, they may be of use for the development of agents against inflammatory and degenerative conditions.

## 1. Introduction

Inflammation is a key factor in various metabolic and cardiovascular disorders. Inflammatory and metabolic diseases are interconnected, e.g., NF-κB signaling is associated with insulin resistance and atherosclerosis. Furthermore, increased circulating factors such as glucose and fatty acids may account for the propagation of inflammatory signals [1,2]. In addition, neurodegenerative disorders are characterized by inflammatory stimuli and the activation of the innate immune system response in the central nervous system (CNS) [3].

The imbalance between oxidative versus antioxidant processes in favor of the former may result in the interruption of cellular homeostasis and an increase in cellular dysfunction, affecting many pathobiochemical markers and paths [4]. Inflammatory agonists such as IL-1β utilize reactive oxygen species (ROS) as part of their signaling, and factors such as NF-κB may be affected by oxidative stress [5,6], making the interrelation between oxidative stress and inflammation obvious. 

ROS may deteriorate the vascular tone in response to metabolic conditions such as hyperglycemia, insulin resistance or hyperlipidemia. They may also disturb endothelial nitrogen monoxide synthetase (eNOS), leading to apoptosis and NOS decoupling. Thus, increased production of superoxide anion radical, peroxynitrite and deficiency of tetrahydrobiopterin impair the vascular relaxation and function [7]. In parallel, atherosclerotic plaque enhances cellular oxidation by macrophages, via the consumption of blood lipids such as oxidized low-density lipoprotein [8]. Hypercholesterolemia has been related to downregulation of antioxidant enzymes and tissue levels of endogenous antioxidants, whilst hypolipidemic agents seem to attenuate the oxidative conditions that may deteriorate during oxidative or nitrosative stress [9].

Classical non-steroidal anti-inflammatory drugs (NSAIDs) are widely applied in inflammatory conditions. However, their non-selective cyclooxygenase (COX)-1 and -2 inhibition results in gastroduodenal irritation [10]. These undesired effects seem to be mitigated via molecular modifications that offer antioxidant capacity to the NSAID molecule [11]. Although selective COX-2 inhibitors reduce the incidence of gastropathy, the increased ratio of thromboxanes towards prostacyclin limits their application in the case of cardiovascular disorders [12]. COX inhibition may lead to the induction of the lipoxygenase (LOX) pathway for the metabolism of arachidonic acid; thus, concomitant LOX inhibition may diminish these effects, increasing the pleiotropic activity of such dual inhibitors [13]. 

The cytoprotective and antiatherogenic effects of L-serine in cell cultures and hypercholesterolemic rabbits have been shown and may be related to the antioxidant effects of this amino acid, via elevation of agents such as nuclear factor-erythroid 2 (NF-E2)-related factor 2 (Nrf2), heme oxygenase-1 (HO-1) and nitrogen monoxide, regulating vascular function and endothelial cell death [14,15]. L-serine has also exerted neuroprotective activity in mice and humans, reducing the formation of the neurotoxic deoxysphinganine and deoxymethylsphinganine [16]. Furthermore, serine participates in the biosynthesis of glycine, an inhibitory neurotransmitter that may contribute to the decrease in neuronal excitotoxicity and seizure induction [17,18]. Moreover, the symptoms of 3-phosphoglycerate dehydrogenase (*3*-*PGDH*) deficiency (attenuation of white matter volume, psychomotor retardation and hypomyelination) seem to be reversed by the oral supplementation of serine [19]. 

Cinnamic acid and cinnamalaldehyde exert antioxidant, anti-inflammatory and cytoprotective activity, increasing NO synthase and decreasing COX-2 and NF-kB expression [20,21]. Furthermore, trans-cinnamic acid bears an anti-obesity effect on high-fat diet-fed mice, improving their lipid profile (reduced total cholesterol and triglyceride levels together with LDL cholesterol) and adipose tissue hypertrophy [22]. Butylated hydroxytoluene (BHT) is an antioxidant, and some of its derivatives possess multiple biological activities [23]. Darbufelone and hybridized molecules bearing the BHT molecular characteristics seem to offer antioxidant, anti-inflammatory, anti-edematogenic and anti-nociceptive potency [24].

In the same direction, vitamin E and its derivative Trolox offer protection in various pathobiological conditions via antioxidant and non-antioxidant activities [25]. We have shown that derivatives of Trolox, (*E*)-3-(3,5-di-*tert*-butyl-4-hydroxyphenyl)acrylic acid and 3,5-di-*tert*-butyl-4-hydroxybenzoic acid were potent hypolipidemic, antioxidant and lipoxygenase inhibitory agents [26].

**Figure 1:**
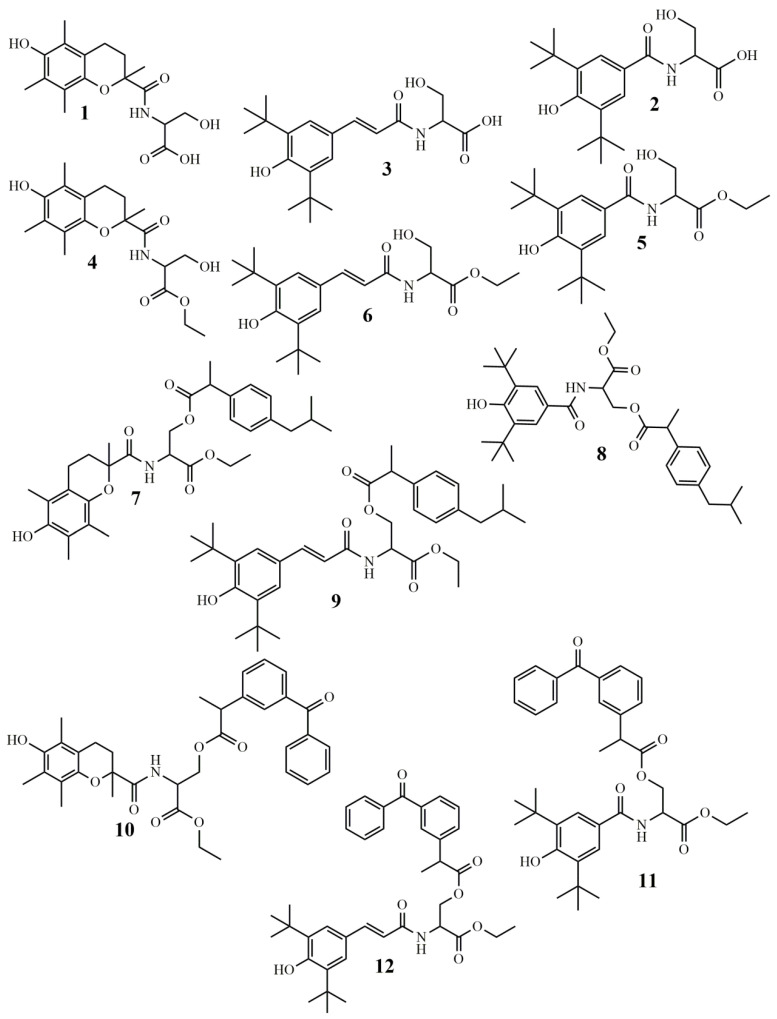
The synthesized novel L-serine derivatives.

**Figure 2:**
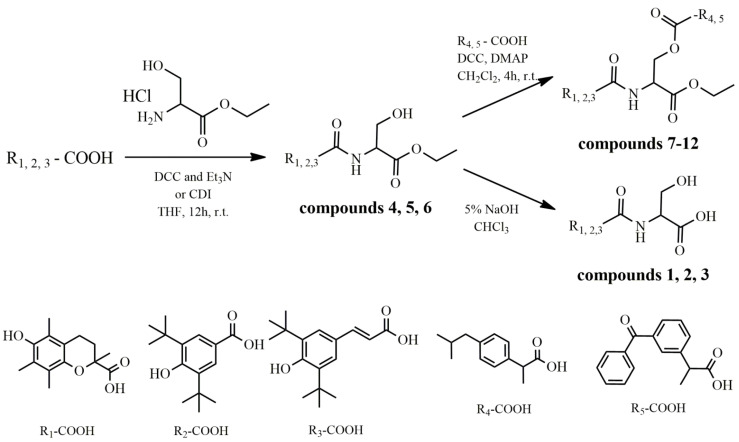
Synthesis of the compounds. DCC: *N*,*N*′-dicyclohexylcarbodiimide; CDI: carbonyldiimidazole; DMAP: *N*,*N*-dimethylaminopyridine; Et_3_N: triethylamine; THF: tetrahydrofuran; r.t: room temperature.

In view of all the above, we considered it of interest to combine L-serine with antioxidant and anti-inflammatory agents. Therefore, we synthesized a series of L-serine and L-serine ethyl ester analogues (Figure 1) amidated with Trolox (R_1_-COOH), 3,5-di-*tert*-butyl-4-hydroxybenzoic acid (R_2_-COOH) and (*E*)-3-(3,5-di-*tert*-butyl-4-hydroxyphenyl)acrylic acid (R_3_-COOH) (Figure 2), yielding compounds **1**–**6**. The methyl esters were further esterified via serine hydroxyl with ibuprofen (R_4_-COOH) and ketoprofen (R_5_-COOH), yielding compounds **7**–**12**. The antioxidant activity of these compounds, expressed as inhibition of rat hepatic microsomal membrane lipid peroxidation, as well as their interaction with the stable radical DPPH, was evaluated. Their anti-inflammatory effect in vivo, as carrageenan-induced rat paw edema inhibition, and in vitro, as inhibition of soybean lipoxygenase, was examined. The hypolipidemic effect of selected derivatives was also tested in vivo. 

## 2. Results

### 2.1. Synthesis

Compounds **4**–**6** were synthesized by direct amidation of L-serine hydrochloride with the respective acid (**R_1_-COOH**, **R_2_-COOH** and **R_3_-COOH**), via the formation of anhydride with DCC (*N*,*N*′-dicyclohexyl-carbodiimide) or the intermediate imidazole derivative using CDI (carbonyldiimidazole), with excellent yields (82–91%). In the case of Trolox (**R_1_COOH**), usage of DCC resulted in a bulky intermediate anhydride, leading to lower yields. The corresponding products were hydrolyzed with sodium hydroxide, yielding compounds **1**–**3**, or esterified with ibuprofen (**R_4_-COOH**) and ketoprofen (**R_5_-COOH**), yielding compounds **7**–**9** and **10**–**12**, respectively. The esterification reaction was carried out using DCC and DMAP with yields varying from 70 to 91%. In total, the lower yield was obtained with the Trolox derivatives, possibly due to steric hindrance. 

### 2.2. Antioxidant and Radical Scavenging Activity

The effect of the new compounds and Trolox on rat hepatic microsomal membrane lipid peroxidation, expressed as IC_50_ values after 45 min of incubation, their clog*P* and total polar surface area (TPSA) values are shown in Table 1. For the acids **1**, **2** and **3**, log*D* values were calculated.

**Figure 3:**
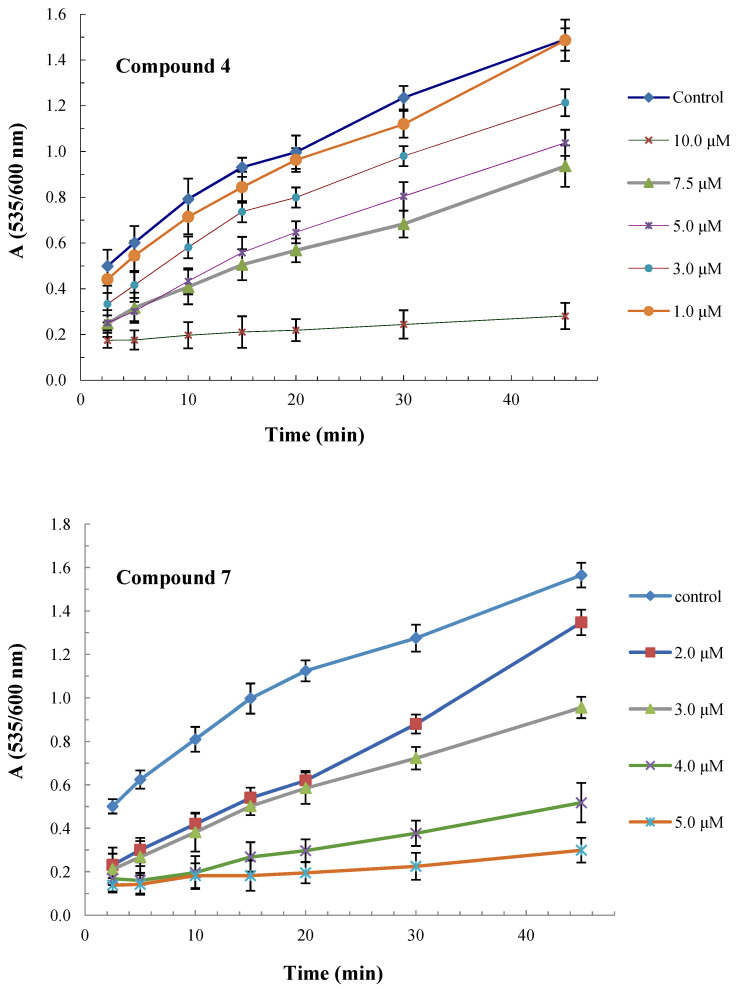
Time course of lipid peroxidation as affected by various concentrations of compounds **4** and **7**.

The time course of lipid peroxidation, as affected by various concentrations of compounds **4** and **7**, is shown in Figure 3.

The interaction of compounds with the lipophilic N-centered 1,1-diphenyl-2-picrylhydrazyl (DPPH) stable free radical is another assay for evaluation of the antioxidant and reducing activity of compounds. The interaction of compounds **1**, **3**, **4**, **6**, **7**, **8**, **9**, **10**, **12** and **Trolox**, at various concentrations, with DPPH is presented in Table 2.

Compounds **2**, **5** and **11** demonstrated lower than 10% inhibition at 200 μΜ.

### 2.3. In Vivo Anti-Inflammatory Activity

The effect of the synthesized compounds on acute inflammation, applying the carrageenan paw edema model, as well as the anti-inflammatory activity of ibuprofen and ketoprofen, used as reference compounds, is shown in Table 3.

### 2.4. Lipoxygenase Inhibitory Activity

The ability of compounds **1**–**12** to inhibit lipoxygenase, presented as IC_50_ values or percent inhibition at 100 μM, towards soybean lipoxygenase 1-B, using linoleic acid as a substrate, after 7 min of incubation, is demonstrated in Table 4. The IC_50_ of nordihydroguaiaretic acid (NDGA), an antioxidant compound acting as a nonspecific inhibitor of lipoxygenase, together with ibuprofen, ketoprofen and BHT, is also included as a reference.

### 2.5. Hypolipidemic Effect

The anti-dyslipidemic activity of compound **4**, the most active non-NSAID containing antioxidant derivative, and compounds **5**, **9**, **11** and **12**, the most active anti-inflammatory compounds, was tested on Triton-induced hyperlipidemia in rats. Simvastatin was used as a reference compound. Results are shown in Table 5.

## 3. Discussion

### 3.1. Antioxidant and Radical Scavenging Activity

Reactive nitrogen and oxygen species participate in the promotion of atherogenesis, mitochondrial dysfunction and protein aggregation, resulting in vascular and neural degeneration [27,28]. The most active antioxidants were the Trolox derivatives. Compound **10** is almost nine times more active than the parent antioxidant acid. The increased activity could be explained by the extended conjugation of the chroman ring, resulting in radical stabilization, after the phenolic hydrogen abstraction. This effect may be further enhanced by the high lipophilicity of compounds **4**, **7** and **10**, compared with Trolox, which permits an effective approach to the lipid phase. For the carboxylic acids **1**, **2** and **3**, log*D* (the octanol/water partition coefficient at pH 7.4) rather than log*P* is preferred for the correlation with their antioxidant activity. Thus, although Trolox derivative **4** is more active than **6**, compound **1** is considerably less active than **3**, probably due to the pronounced difference in their log*D* values. The low Log*D* value and the less extended conjugation may be the reason for the inactivity of compound **2**.

Ιbuprofen and ketoprofen derivatives seem to offer similar antioxidant activity depending on the relevant antioxidant moiety of the compound. The ethylated serine structures **5** and **6** had the highest activity in comparison to the other 3,5-di-*tert*-butyl-4-hydroxybenzoic acid and (*E*)-3-(3,5-di-*tert*-butyl-4-hydroxyphenyl) acrylic acid derivatives.

Compounds **8**, **9** and **12** exhibited the same level of antioxidant activity with increasing concentrations. This may be due to their high lipophilicity leading to poor solubility in the experimental medium, and to difficulty in the IC_50_ determination. However, both (*E*)-3-(3,5-di-tert-butyl-4-hydroxyphenyl)acrylic derivatives **9** and **12** seem to be more potent than **8** or **11**. Additionally, this is the case for derivatives **5** and **6**. The antioxidant activity of **6** was almost seven times higher, compared with the 3,5-di-tert-butyl-4-hydroxybenzoic acid derivative **5**. This difference in the activity is confirmed by our previous results of compounds bearing these acids, and the equal inhibition in various concentrations of the compounds seems to be related to their physicochemical properties and not only to their antioxidant capacity [26,29].

In the DPPH radical scavenging experiment, performed in a homogenous reaction mixture, the reducing activity of Trolox derivatives (**1**, **4**, **7**, **10**) is about the same as with Trolox. Similarly, compounds **3**, **6**, **9** and **12** were potent reducing agents, whereas, in the lipid peroxidation assay, their high lipophilicity intervened with the assay performed in an aqueous medium. 3,5-Di-*tert*-butyl-4-hydroxybenzoic acid derivatives **2**, **5**, **8** and **11** were of low activity, partly in agreement (with the exception of compound **5**) with their weak activity in the lipid peroxidation experiments.

### 3.2. In Vivo Anti-Inflammatory Activity

Carrageenan-induced paw edema is a well-known and widely used biphasic model of acute inflammation. In the delayed phase, more than one hour after administration, neutrophil infiltration, prostaglandin production and release of pro-inflammatory cytokines are involved [30].

All compounds could reduce paw edema from about 30 to 67%, at 150 μmol/kg, i.p., 3.5 h after carrageenan administration. Hydrolyzed and ethylated serine derivatives **1**–**6** were of similar activity according to their corresponding antioxidant moiety, with the exception of compound **6** that shows a remarkable and statistically significant improvement in inhibition compared with compound **3**. Ibuprofen derivatives showed partly better (but not statistically significant) activity than the non-NSAID esterified compounds but were of lower potency than those of ketoprofen. However, with the exception of compound **10**, all the NSAID derivatives were more active than the parent NSAID alone. This may indicate that the compounds act without prior hydrolysis to the parent NSAID. Apart from the antioxidant and reducing potential of the compounds, it seems that amidation with serine may add to the appearance of anti-inflammatory activity in the antioxidant acids. We have previously reported that esters or amides of ibuprofen and ketoprofen enhanced the anti-inflammatory activity of the parent molecules [31,32], and that antioxidant acids such as Trolox amidated with L-cysteine or cysteamine yielded potent anti-inflammatory agents [29]. Unlike butylated hydroxytoluene which is devoid of anti-inflammatory activity [23], 3,5-di-*tert*-butyl-4-hydroxybenzoic acid derivatives possess significant anti-inflammatory activity, not always related to the antioxidant activity of the compounds, a finding that may partly be related to the lipoxygenase inhibitory activity of the compounds [23,26,29,31,33]. Additionally, since oxidative stress plays a critical role in inflammatory processes, compounds bearing the 3,5-di-*tert*-butyl-4-hydroxybenzyl moiety may offer considerable anti-inflammatory activity [34,35]. Furthermore, this moiety has been shown to mitigate the production of various pro-inflammatory mediators such as IL-1b and TNF-a by decreasing the amount of various activating oxidant factors such as myeloperoxidase in neutrophils and various other immune cells [24]. Similarly, in this report, 3,5-di-*tert*-butyl-4-hydroxyphenyl derivatives, and especially compound **11**, demonstrate an interestingly increased anti-inflammatory activity, especially if the low antioxidant capacity of these compounds is considered.

### 3.3. Lipoxygenase Inhibitory Activity

Lipoxygenases form the second major path of arachidonic acid metabolism, involved in the synthesis of leukotrienes that are implicated in various disorders, e.g., asthma, arthritis, atherosclerosis and neurodegenerative and autoimmune diseases. Furthermore, there is a lack of clinical LOX inhibitors, with the drawback of adverse effects restricting their approval [36]. Soybean lipoxygenase, having structural and functional similarities with mammalian lipoxygenases, is often used for the study of anti-inflammatory agents [37].

The majority of the compounds presented moderate activity in this experiment. The IC_50_ values of some compounds could not be calculated, due to their low solubility in the reaction mixture. Trolox derivatives showed very low potency, as expected, considering that Trolox itself has an IC_50_ higher than 300 μΜ, in combination with the increased rigidity and bulk of the corresponding compounds, especially that of **7** and **10**. Di-*tert*-butylphenol derivatives **2**, **3**, **5** and **6** had similar activity to butylated hydroxytoluene. Ibuprofen and ketoprofen derivatives **8** and **11** were more active than the NSAID or BHT alone. It has been previously shown [26,38] that this structural 3,5-di-*tert*-butyl-4-hydroxyphenyl moiety may contribute to increased lipoxygenase inhibitory activity. Compound **8** demonstrated surprisingly high activity, several times higher than the parent compounds ibuprofen and compound **5**, pointing out that they act as a whole molecular entity in LOX inhibition. Ibuprofen derivatives **8** and **9** were more active than ketoprofen derivatives **11** and **12**. This may partly be related to the less rigid structure of the former NSAID, since we have shown that rigid molecules seem to offer diminished activity [39,40]. 4-Hydroxy cinnamic acid shows low potency in LOX inhibition; however, compounds bearing this structure or the (*E*)-phenyl-propene moiety show considerable activity on LOX inhibition [41,42].

When linoleic acid was used at 1 mM, a concentration higher than the saturating substrate concentration, no inhibition was observed, under the same experimental conditions, indicating competitive inhibition of lipoxygenase by the compounds.

### 3.4. Hypolipidemic Effect

Administration of Triton-WR1339 to rats leads to a sharp increase in plasma cholesterol and especially triglyceride levels, after 24 h, and a decrease in the above indices seems to partly interfere with cholesterol biosynthesis [43]. Simvastatin was used as a reference compound, since statins, apart from highly active hypolipidemic agents via 3-hydroxy-3-methylglutaryl coenzyme A (HMG-CoA) reductase inhibition, possess multiple actions such as antioxidant ability and eNOS activity [44].

Inflammation and oxidative stress are closely related to hyperlipidemia. Hypercholesterolemia and oxidized LDL cholesterol are the main contributors to the development of atherosclerosis and the progression of neurodegenerative disorders such as Alzheimer’s disease [27,45]. Furthermore, we have shown that amide derivatives of NSAIDs and antioxidants possess hypolipidemic properties [23,26,29,32]. Compounds with dual anti-inflammatory and antioxidant potential may improve the cardiovascular state of the patient via various mechanisms such as NF-κB reduction and Nrf2 amplification, together with the reduction in ox-LDL levels [46]

All compounds reduced lipidemic indices considerably, although triglyceride reduction was less pronounced, whilst there were not significant differences between our tested compounds with the exception of the effect of compound **9** on LDL cholesterol levels. The relatively decreased activity on triglycerides may be related to the more profound increase in triglycerides by Triton administration. Compound **9** was the most active with similar results in total and LDL cholesterol with simvastatin. The tested compounds effectively reduced triglyceride levels, while simvastatin had no effect. These results may be related to the antioxidant and the NSAID moiety. Antioxidant and anti-inflammatory activity may contribute to this effect in parallel or separately. Thus, compound **4** had low activity in carrageenan edema inhibition, and compound **5** had a modest antioxidant capacity, but both produced a significant hypolipidemic effect. However, the dual antioxidant and anti-inflammatory activity may offer a more powerful combination as is the case with compound **9**.

## 4. Materials and Methods

### 4.1. General

All commercially available chemicals were of the appropriate purity. IR spectra were recorded on a Perkin Elmer Spectrum BX FT-IR spectrometer. ^1^H NMR and ^13^C NMR spectra were recorded using a BRUKER Avance III-300 MHz or an AGILENT DD2-500 MHz spectrometer. All chemical shifts are reported in δ (ppm), and signals are given as follows: s, singlet; d, doublet; t, triplet; m, multiplet. Melting points (mp) were determined with a MEL-TEMPII (Laboratory Devices, USA) instrument and are uncorrected. The microanalyses were performed on a Perkin-Elmer 2400 CHN elemental analyzer.

κ-Carrageenan and lipoxygenase type I-B from soybean were purchased from Sigma. For the in vivo experiments, Wistar rats (160–220 g, 3–4 months old) were kept in the Centre of the School of Veterinary Medicine (EL54 BIO42), Aristotelian University of Thessaloniki, which is registered by the official state veterinary authorities (presidential degree 56/2013, in harmonization with the European Directive 2010/63/EEC). The experimental protocols were approved by the Animal Ethics Committee of the Prefecture of Central Macedonia (no. 270079/2500). The statistical analysis of the results was performed using GraphPad Software (version 7).

### 4.2. Synthesis

#### 4.2.1. General Methods for the Synthesis of Compounds **4**–**6**

For compounds **5** and **6**, in a suspension of serine ethyl ester hydrochloride (1.2 mmol) in tetrahydrofuran (10 mL), triethylamine (Et_3_N) (1.4 mmol) was added, and after 5 min, the corresponding acid (1 mmol) and *N*,*N*′-dicyclohexylcarbodiimide (DCC, 1.3 mmol) were added. After stirring for 12 h at room temperature, the precipitated material was filtered off, and THF was evaporated. The resulting mixture was dissolved in CH_2_Cl_2_ (10 mL), successively washed with water and 5% NaHCO_3_ solution and dried (Na_2_SO_4_), and the final compounds were isolated with flash chromatography using petroleum ether and ethyl acetate as eluents.

For compound **4**, the acid (1 mmol) was dissolved in THF (10 mL), and a solution of carbonyldiimidazole (CDI, 1.05 mmol), in the same solvent, was added. After 45 min, the solution was poured into a suspension of the amine (1.2 mmol) in THF (5 mL), and the mixture was left for 12 h with stirring at room temperature. Then, THF was evaporated, the resulting mixture was dissolved in CH_2_Cl_2_ (10 mL), successively washed with water and 5% NaHCO_3_ solution and dried (Na_2_SO_4_) and the final compounds were isolated with flash chromatography using petroleum ether and ethyl acetate as eluents.

#### 4.2.2. General Method for the Synthesis of Compounds **1**–**3**

The corresponding ethyl ester amides (3 mmol) were dissolved in CHCl_3_ (10 mL), 5 mL of a 5% aqueous NaOH solution was added and the mixture was left for 1 h with stirring at room temperature. Then, CHCl_3_ and the produced ethanol were evaporated, the resulting mixture was dissolved in CHCl_3_ (10 mL) and the aqueous solution was acidified with aqueous solution of 5% HCl. The aqueous phase was extracted three times with 20mL CHCl_3_ and dried (Na_2_SO_4_), and the final compounds were purified with recrystallization from acetone and petroleum ether.

#### 4.2.3. General Method for the Synthesis of Compounds **7**–**12**

The acid (1 mmol) was dissolved in CH_2_Cl_2_ (20 mL), the corresponding serine derivative (1.05 mmol), *N*,*N*′-dicyclohexylcarbodiimide (DCC, 1.3 mmol) and 4-(dimethylamino)pyridine (DMAP, 0.1 mmol), in the same solvent, were added and the mixture was left for 4 h with stirring at room temperature. Then, the resulting mixture was filtered, and the final compounds were isolated with flash chromatography using petroleum ether and ethyl acetate as eluents.

*3-Hydroxy-2-(6-hydroxy-2,5,7,8-tetramethylchroman-2-carboxamido)propanoic acid*, **1**, White solid, yield 76%, m.p. 50–75 °C. IR (Nujol) λ_max_: 3410 (O-H), 3179 (N-H), 3500–2500 (O-H carboxylic acid), 1735, 1726 (C=O carboxylic acid LD, LL), 1655, 1648 (C=O amide LD, LL) cm^−1^. ^1^H NMR (CDCl_3_ + DMSO-*d*_6_) δ(ppm): 1.19 (s, 3H, C1 chromane -CH_3_), 1.71–1.59 (m, 1H, C2 chromane axial), 1.89, 1.87, 1.80 (3s, 9H, -CH_3_ aromatic), 2.05–1.90 (m, 1H, C2 chromane equatorial), 2.3 (s, 2H, C3 chromane), 3.71–3.51 (m, 2H, HOCH_2_-), 4.13 (ddd, 1H, *J* = 11.3, 7.7, 3.4 Hz, asymmetric C). Anal. Calcd for C_17_H_23_ΝO_6_: C, 60.52; H, 6.87; Ν, 4.15. Found: C, 60.22; H, 7.16; Ν, 4.19.

*2-(3,5-Di-tert-butyl-4-hydroxybenzamido)-3-hydroxypropanoic acid*, **2**, White solid, yield 84%, m.p. 185 °C. IR (Nujol) λ_max_: 3624 (O-H aromatic), 3342 (N-H), 3300–2500 (O-H), 1718 (C=O acid), 1631 (C=O amide), 1602, 1584 (C-C aromatic) cm^−1^. ^1^H NMR (CDCl_3_ + DMSO-*d*6) δ(ppm): 1.38 (s, 18H, -CH_3_), 3.91 (ddd, 2H, *J* = 14.5, 11.4, 3.6 Hz, HOCH_2_-), 4.64 (bs, 1H, asymmetric C H), 7.60 (s, 2H, aromatic H). Anal. Calcd for C_18_H_27_ΝO_5_: C, 64.07; H, 8.07; Ν, 4.15. Found: C, 63.99; H, 7.94; Ν, 4.12.

*(E)-2-(3-(3,5-di-tert-butyl-4-hydroxyphenyl)acrylamido)-3-hydroxypropanoic acid*, **3**, White solid, yield 89%, m.p. 95–119 °C. IR (Nujol) λ_max_: 3628 (O-H), 3314 (N-H), 3300–2500 (O-H carboxylic acid), 1736 (C=O carboxylic acid), 1654 (C=O amide), 1593 (C-C aromatic) cm^−1^. ^1^H NMR (CDCl_3_ + DMSO-*d*6) δ(ppm): 1.37 (s, 18H, -CH_3_), 4.21–3.88 (m, 2H, HOCH_2_-), 4.73 (s, 1H, asymmetric C H), 5.47 (s, 1H, O-H), 6.53 (d, 1H, *J* = 15.5 Hz, -CH=CH-C=O), 7.32 (s, 2H, aromatic H), 7.56 (s, 1H, N-H), 7.63 (d, 1H, *J* = 15.5 Hz, -CH-CH-C=O). Anal. Calcd for C_20_H_29_ΝO_5_: C, 66.09; H, 8.04; Ν, 3.85. Found: C, 65.87; H, 7.71; Ν, 3.55.

*Ethyl 3-hydroxy-2-(6-hydroxy-2,5,7,8-tetramethylchroman-2-carboxamido)propanoate*, **4**, Flash chromatography (petroleum ether/ethyl acetate 3:2). White solid, yield 82%, m.p. 73–83 °C. IR (Nujol) λ_max_: 3501 (O-H phenolic), 3417, 3404 (N-H LL and LD), 3600–3200 broad (O-H alcoholic), 1740 (C=O ester), 1651 (C=O amide), 1515 (C-C aroamatic) cm^−1^. ^1^H NMR (CDCl_3_), δ (ppm): 1.36–1.18 (m, 3H, -OCH_2_CH**_3_**), 1.58, 1.57 (s, 3H, C1 chromane-CH_3_), 2.02–1.90 (m, 1H, C2 chromane axial), 2.21 and 2.12 (s, 6H, -CH_3_ aromatic), 2.26 (d, 3H, *J* = 4.7 Hz, -CH_3_ aromatic), 2.48–2.29 (m, 1H, C2 chromane equatorial), 2.72–2.59 (m, 2H, C3 chromane), 3.86–3.71 (m, 2H, -CH-CH_2_-OH), 3.99 (d, 1H, *J* = 4.0 Hz, -CH_2_-OH), 4.33–4.11 (m, 2H, C-O-CH_2_-CH_3_), 4.56 (ddd, 1H, *J* = 10.8, 7.1, 4.0 Hz, -CH-CH_2_-OH), 7.51–7.35 (m, 1H, -NH-). Anal. Calcd for C_19_H_27_ΝO_6_: C, 62.45; H, 7.45; Ν, 3.83. Found: C, 62.08; H, 7.11; Ν, 4.02.

*Ethyl 2-(3,5-di-tert-butyl-4-hydroxybenzamido)-3-hydroxypropanoate*, **5**, Flash chromatography (petroleum ether/ethyl acetate 2:1). White solid, yield 91%, m.p. 54–56 °C. IR (Nujol) λ_max_: 3569 (O-H), 3343 (N-H), 1740 (C=O ester), 1628 (C=O amide), 1600, 1532 (C-C aromatic) cm^−1^. ^1^H NMR (CDCl_3_), δ (ppm): 1,4 (t, 2H, *J* = 7.0 Hz, -OCH_2_CH_3_), 1,5 (s, 18H, -CH3), 4.09 (qd, 1H, *J* = 11.0, 3.4 Hz, HOCH_2_-C), 4.32 (q, 1H *J* = 7.0 Hz, -OCH_2_CH_3_), 4.85 (s, 1H, asymmetric C H), 5.60 (s, 1H, phenolic-OH), 6.99 (s, 1H, -NH), 7.69 (s, 2H, aromatic H). Anal. Calcd for C_20_H_31_ΝO_5_: C, 65.73; H, 8.55; Ν, 3.83. Found: C, 65.33; H, 8.91; Ν, 3.70.

*(E)-ethyl 2-(3-(3,5-di-tert-butyl-4-hydroxyphenyl)acrylamido)-3-hydroxypropanoate*, **6**, Flash chromatography (petroleum ether/ethyl acetate 2:1 and subsequently 1:1). Pale yellow solid, yield 90%, m.p. 72–83 °C. IR (Nujol) λ_max_: 3583 (O-H), 3297 (N-H), 1738 (C=O ester), 1654 (C=O amide), 1594 (C-C aromatic) cm^−1^. ^1^H NMR (CDCl_3_) δ(ppm): 1.35 (t, 3H, *J* = 7.0 Hz, -OCH_2_CH_3_), 1.46 και 1.48 (s, *18H*, -CH_3_), 4.03–4.07 (m, 2H, HOCH_2_-), 4.31 (q, 2H, *J* = 7.0 Hz, -OCH_2_CH_3_), 4.88–4.77 (m, 1H, asymmetric C H), 5.48 (s, 1H, phenolic-OH), 6.38 (d, 1H, *J* = 15.5 Hz, -CH=CH-C=O), 6.56 (d, 1H, *J* = 5.8 Hz, -NH), 7.38 (s, 2H, aromatic H), 7.63 (d, 1H, *J* = 15.5 Hz, -CH-CH-C=O). Anal. Calcd for C_22_H_33_ΝO_5_: C, 67.49; H, 8.50; Ν, 3.58. Found: C, 67.31; H, 8.60; Ν, 3.28.

*Ethyl 2-(6-hydroxy-2,5,7,8-tetramethylchroman-2-carboxamido)-3-((2-(4-isobutylphenyl)propanoyl)oxy)propanoate*, **7**, Flash chromatography (petroleum ether/ethyl acetate 3:1). Viscous transparent oil, yield 78%, IR λ_max_: 3600–3100 broad (O-H and N-H), 173090, 3049 (C-H aromatic), 2977, 2956, 2934 (C-H alkyl), 1741 (C=O ester), 1666 (C=O amide), 1512, 15454 (C-C aromatic) cm^−1^. ^1^H NMR (CDCl_3_) δ(ppm): 0.93–0.84 (m, 6H, CH_3_-CH-CH_3_), 1.56–1.05 (m, 9H, C1 chromane-CH_3_, -CO-CH-CH_3_ and -O-CH_2_-CH_3_), 2.00–1.78 (m, 2H, C2 chromane axial, CH_3_-CH-CH_3_), 2.32–2.05 (m, 10H, -CH_3_ aromatic and C2 chromane equatorial), 2.48–2.33 (m, 2H, C3 chromane), 2.73–2.51 (m, 2H, -CH_2_CH-(CH_3_)_2_),), 3.73–3.31 (m, 1H, -CO-CH-CH_3_), 4.21–3.89 (m, 2H, -O-CH_2_CH-NH), 4.57–4.23 (m, 2H, -O-CH_2_-CH_3_), 4.75–4.63 (m, 1H, O-CH_2_CH-NH), 5.33 (s, 1H, -OH), 7.20–6.96 (m, 4H, ibuprofen aromatic H). Anal. Calcd for C_32_H_43_ΝO_7_: C, 69.42; H, 7.83; Ν, 2.53. Found: C, 69.29; H, 7.77; Ν, 2.30.

*Ehyl 2-(3,5-di-tert-butyl-4-hydroxybenzamido)-3-((2-(4-isobutylphenyl)propanoyl)oxy)propanoate*, **8**, Flash chromatography (petroleum ether/ethyl acetate 6:1 and subsequently 5:1). White solid, yield 92%, m.p. 55–59 °C. IR (Nujol) λ_max_: 3583, 3444 (O-H LL and LD), 3332 (N-H), 1746, 1731 (C=O ester, LL and LD), 1656, 1632 (C=O amide, LL and LD), 1584, 1562 (C-C aromatic) cm^−1^. ^1^H NMR (CDCl_3_) δ(ppm): 0.90, 0.88 (d, 6H, *J* = 7.1 Hz, CH_3_-CH-CH_3_), 1.27, 1.18 (t, 3H, *J* = 7.1 Hz, -O-CH_2_-CH_3_), 1.45–1.35 (m, 3H, -CO-CH-CH_3_), 1.50, 1.49 (s, 18H, -C(CH_3_)_3_), 1.91–1.73 (m, 1H, CH_3_-CH-CH_3_), 2.43, 2.42 (d, 2H, *J* = 7.2 Hz, -CH_2_CH-(CH_3_)_2_), 3.78–3.67 (m, 1H, -CO-CH-CH_3_), 4.28–3.97 (m, 2H, -O-CH_2_CH-NH), 4.7–4.4 (m, 2H, -O-CH_2_-CH_3_), 5.05–4.95 (m, 1H, O-CH_2_CH-NH), 5.63 (s, 1H, phenolic-OH), 6.89, 6.82 (d, 1H, *J* = 7.2 Hz, -NH-), 7.06 (d, 2H, *J* = 7.9 Hz, aromatic C3, C5 phenyl H), 7.17 (d, 2H, *J* = 7.9 Hz, aromatic C2, C6 phenyl H), 7.66, 7.63 (s, 2H, aromatic di-*tert*-butyl phenyl H). Anal. Calcd for C_33_H_47_ΝO_6_: C, 71.58; H, 8.56; Ν, 2.53. Found: C, 71.48; H, 8.58; Ν, 2.74.

*(E)-ethyl 2-(3-(3,5-di-tert-butyl-4-hydroxyphenyl)acrylamido)-3-((2-(4-isobutylphenyl)propanoyl)oxy)propanoate*, **9**, Flash chromatography (petroleum ether/ethyl acetate 5:1). White solid, yield 79%, m.p. 51–60 °C. IR (Nujol) λ_max_: 3628 (O-H), 3268 broad (N-H), 1746 (C=O ester), 1655 (C=O amide), 1595, 1531 (C-C aromatic) cm^−1^. ^1^H NMR (CDCl_3_), δ (ppm): 0.91–0.83 (m, 1H, CH_3_-CH-CH_3_), 1.27, 1.15 (t, 3H, *J* = 7.1 Hz, -O-CH_2_-CH_3_), 1.49 (s, 18H, -C(CH_3_)_3_), 1.85–1.65 (m, 3H, -CO-CH-CH_3_), 2.16–2.06 (m, 1H, CH_3_-CH-CH_3_), 2.41 (dd, 2H, *J* = 7.1, 2.2 Hz, -CH_2_CH-(CH_3_)_2_),), 3.78–3.64 (m, 1H, -CO-CH-CH_3_), 4.25–3.92 (m, 2H, -O-CH_2_CH-NH), 4.63–4.35 (m, 2H, -O-CH_2_-CH_3_), 5.02–4.93 (m, 1H, O-CH_2_CH-NH), 6.34–6.06 (m, 2H, -CH=CH-C=O and -NH-), 7.10 (dd, 2H, *J* = 8.0, 3.3 Hz, aromatic C3, C5 phenyl H), 7.20 (dd, 2H, *J* = 8.0, 2.2 Hz, aromatic C2, C6 phenyl H), 7.38, 7.37 (s, 2H, aromatic di-*tert*-butyl phenyl H), 7.59 (dd, 1H, *J* = 15.6, 6.1 Hz, -CH-CH-C=O). Anal. Calcd for C_35_H_49_ΝO_6_: C, 72.51; H, 8.52; Ν, 2.42. Found: C, 72.09; H, 8.27; Ν, 2.44.

*Ethyl 3-((2-(3-benzoylphenyl)propanoyl)oxy)-2-(6-hydroxy-2,5,7,8-tetramethylchroman-2-carboxamido)propanoate*, **10**, Flash chromatography (petroleum ether/ethyl acetate 3:1 and subsequently 2:1). Semifluid oil, yield 70%. IR (Nujol) λ_max_: 3418 (O-H phenol), 3327 (N-H), 3600–3100 broad (O-H alcohol), 1740 (C=O ester), 1660, 1626 (C=O amide diastereomers), 1597, 1580 (C-C aromatic) cm^−1^. ^1^H NMR (CDCl_3_), δ (ppm): 1.31–1.16 (m, 3H, -O-CH_2_-CH_3_), 1.44–1.32 (m, 3H, C1 chromane-CH_3_), 1.59–1.47 (m, 3H, -CO-CH-CH_3_), 1.92–1.83 (m, 1H, C2 chromane axial), 2.20–2.10 (m, 9H, -CH_3_ aromatic), 2.35–2.25 (m, 1H, C2 chromane equatorial), 2.70–2.50 (m, 2H, C3 chromane), 3.85–3.59 (m, 1H, -CO-CH-CH_3_), 4.23–3.92 (m, 2H, -O-CH_2_CH-NH), 4.57–4.28 (m, 2H, -O-CH_2_-CH_3_), 4.73–4.63 (m, 1H, O-CH_2_CH-NH), 7.84–7.29 (m, 9H, aromatic ketoprofen). Anal. Calcd for C_35_H_39_ΝO_8_: C, 69.87; H, 6.53; Ν, 2.33. Found: C, 69.69; H, 6.47; Ν, 2.11.

*Ethyl 3-((2-(3-benzoylphenyl)propanoyl)oxy)-2-(3,5-di-tert-butyl-4-hydroxybenzamido)propanoate*, **11**, Flash chromatography (petroleum ether/ethyl acetate 5:1 and subsequently 4:1). Viscous transparent oil, yield 91%. IR λ_max_: 3624, 3585 (O-H LL and LD), 3313 (N-H), 1739 broad (C=O ester), 1658 broad (C=O amide), 1599, 1580 (C-C aromatic) cm^−1^. ^1^H NMR (CDCl_3_), δ (ppm): 1.27 (td, 3H, *J* = 7.1, 3.3 Hz, -O-CH_2_-CH_3_), 1.47, 1.46, 2.19, (s, 18H, -C(CH_3_)_3_), 1.56, 1.53 (d, *J* = 3.4 Hz, 3H, -CO-CH-CH_3_), 3.89–3.77 (m, 1H, -CO-CH-CH_3_), 4.26–4.01 (m, 2H, O-CH_2_CH-NH), 4.71–4.46 (m, 2H, -O-CH_2_-CH_3_), 5.03–4.94 (m, 1H, O-CH_2_CH-NH), 5.63 (s, 1H, phenol-OH), 6.9, 6.85 (d, 1H, *J* = 7.2 Hz, -NH-), 7.83–7.35 (m, 11H, aromatic H). Anal. Calcd for C_36_H_43_ΝO_7_: C, 71.86; H, 7.20; Ν, 2.33. Found: C, 71.76; H, 7.14; Ν, 2.51.

*(E)-ethyl 3-((2-(3-benzoylphenyl)propanoyl)oxy)-2-(3-(3,5-di-tert-butyl-4-hydroxyphenyl)acrylamido)propanoate*, **12**, Flash chromatography (petroleum ether/ethyl acetate 6:1 and subsequently 4:1). Yellow solid, yield 81%, m.p. 57–67 °C. IR (Nujol) λ_max_: 3583 (O-H), 3266 (N-H), 1741 (C=O ester), 1655 (C=O ketone), 1619 (C=O amide), 1595 (C-C aromatic) cm^−1^. ^1^H NMR (CDCl_3_) δ(ppm): 1.25, 1.19 (t, 3H, *J* = 7.2 Hz, -O-CH_2_-CH_3_), 1.47 (s, 18H, C(CH_3_)_3_), 1.57 (d, 3H, *J* = 7.2 Hz, -CO-CH-CH_3_), 3.84 (q, 1H, *J* = 7.2 Hz, -CO-CH-CH_3_), 4.25–3.97 (m, 2H, -O-CH_2_CH-NH), 4.64–4.42 (m, 2H, -O-CH_2_-CH_3_), 5.02–4.94 (m, 1H, O-CH_2_CH-NH), 5.46 (s, 1H, -OH), 6.36 (d, 1H, *J* = 15.6 Hz, CH=CH-C=O), 6.44 (dd, 1H, *J* = 16.5, 7.7 Hz, –NH-), 7.37 (s, 2H, aromatic di-*tert*-butyl phenyl H), 7.86–7.41 (m, 10H, aromatic ketoprofen and -CH-CH-C=O). Anal. Calcd for C_38_H_45_ΝO_7_: C, 72.70; H, 7.23; Ν, 2.23. Found: C, 72.53; H, 7.43; Ν, 2.41.

### 4.3. Biological Evaluation

#### 4.3.1. In Vitro Lipid Peroxidation

The peroxidation of the heat-inactivated rat hepatic microsomal fraction was induced by ferrous ascorbate. The studied compounds, in dimethylsulfoxide, were added at concentrations of 1 μM–1 mM. Aliquots were taken from the incubation mixture (37 °C) for 45 min. Lipid peroxidation was assessed spectrophotometrically (535/600 nm) as a 2-thiobarbituric acid reactive material. The incubation mixture and solvents were found not to interfere with the assay [47].

#### 4.3.2. In Vitro Interaction with the Stable Radical 1,1-Diphenyl-2-picrylhydrazyl (DPPH)

Compounds, dissolved in absolute ethanol, at concentrations of 25–200 μM, were added to an equal volume of an ethanolic solution of DPPH (final concentration 200 μM) at room temperature (22 ± 2 °C). Absorbance (517 nm) was recorded after 30 min [26]. For the calculation of percent interaction, the residual absorbance of DPPH Ai (at a fixed concentration) after its interaction with the examined compounds (at various concentrations) was compared with the control absorbance Ao, without the addition of compounds, following the equation:

(1) $$\mathrm{Percent}\;\mathrm{interaction}=100-\left(\frac{\mathrm{Ai}}{\mathrm{Ao}}\times 100\right)$$

#### 4.3.3. Carrageenan-Induced Paw Edema

An amount of 0.1 mL of an aqueous carrageenan solution (1% *w/v*) was injected into the hind paw of adult Wistar rats (6 rats were used for each compound). The tested compounds (in water with a few drops of Tween 80) were administered i.p. (0.15 mmol/kg) 5 min before the carrageenan administration. The produced edema, after 3.5 h, was estimated as paw weight increase [32].

#### 4.3.4. In Vitro Evaluation of Lipoxygenase Activity

The reaction mixture contained the test compounds, dissolved in absolute ethanol (0.1 mL) at various concentrations, or the solvent (control, 0.1 mL), soybean lipoxygenase dissolved in 0.9% NaCl solution (250 u/mL, 0.2 mL) and sodium linoleate (100 μM) in Tris–HCl buffer, pH 9.0 (2.7 mL). The reaction was followed for 7 min at 28 °C, recording the absorbance of a conjugated diene structure at 234 nm, due to the formation of 13-hydroperoxy-linoleic acid. The performance of the assay was checked using nordihydroguaiaretic acid as a reference. For the estimation of the type of inhibition, the above experiments were repeated, using sodium linoleate at 1 mM, which is higher than the saturating substrate concentration [32].

#### 4.3.5. Effect on Plasma Cholesterol and Triglyceride Levels

Hyperlipidemia was induced by the i.p. administration of Triton WR 1339 (200 mg/kg) to adult Wistar rats (6 rats were used for each compound). The examined compounds (0.15 mmol/kg) were administered i.p. one hour later. Blood was taken from the aorta after 24 h, for the determination of plasma total cholesterol, LDL cholesterol and triglyceride concentration, using commercial kits [48].

## 5. Conclusions

In the present study, the described derivatives of antioxidant acids with L-serine, with or without NSAID conjugation, were designed to attain a series of biological properties aiming at the prevention or restoration of various pathological implications of degenerative and inflammatory conditions. L-serine incorporation seems to offer at least equal or several times more improved antioxidant activity, especially in the case of NSAID incorporation on the parent antioxidant amino acid derivatives. However, the 3,5-di-*tert*-butyl-4-hydroxybenzoic acid moiety offers a less effective antioxidant capacity, potentially due to steric and physicochemical reasons. All the compounds may offer considerable anti-inflammatory activity that may, in part, especially in the case of compounds **8** and **11**, be derived from the lipoxygenase inhibitory ability, since they bear a very weak antioxidant effect, whilst the effect of NSAID derivatives and especially of compound **9** may be related to both these actions and to other factors that remain to be clarified. Finally, as for the hypolipidemic activity, the tested compounds may offer an improvement in the lipidemic indices, in comparison to the parent acids, a fact that may also be related to the L-serine moiety incorporation. Compound **9** showed antioxidant and radical scavenging characteristics accompanied by anti-inflammatory, hypolipidemic and especially cholesterol-lowering abilities.

Keeping in mind the adverse effects that may derive from multiple-drug therapy for multicausal diseases, it could be concluded that multifunctional compounds such as compound **9** and some others described in this report may assist in achieving avoidance of drug interactions and better patient tolerance and compliance.

## Figures and Tables

**Table 1:** Effect of the synthesized compounds on rat microsomal membrane lipid peroxidation.

Compound	Inhibition of Lipid Peroxidation IC_50_ (μΜ) ^a^	clog*P* ^b^	TPSA ^b^, Å^2^	log*D* ^b^
**1**	150	2.06	116.09	−2.320
**2**	40%/1 mM	2.90	106.85	−0.563
**3**	80	3.40	106.85	0.161
**4**	7.3	2.75	105.09	
**5**	163	3.69	95.86	
**6**	22	4.21	95.86	
**7**	3.4	7.54	111.17	
**8**	30%/500 μM	8.47	101.94	
**9**	20%/50 μM	8.99	101.94	
**10**	2.8	6.62	128.24	
**11**	30%/1 mM	7.55	119.01	
**12**	20%/100 μM	8.07	119.01	
**Trolox**	25	3.09		

^a^ After 45 min of incubation; Trolox: 6-hydroxy-2,5,7,8-tetramethylchroman-2-carboxylic acid. ^b^ clog*P*: Chem Bio Draw Ultra 12.0; TPSA: Molinspiration Cheminformatics 2021; log*D*: MedChem Designer™. All determinations of lipid peroxidation inhibition of the compounds were performed at least in triplicate, and standard deviation is always within ±10% of the mean value.

**Table 2:** Interaction of compounds **1**, **3**, **4**, **6**, **7**, **8**, **9**, **10**, **12** and **Trolox** at various concentrations, with DPPH (200 μΜ) ^a^.

Compound	Percent Interaction with DPPH			
	200 μΜ	100 μΜ	50 μΜ	25 μΜ
**1**	90.4	87.0	48.0	19.6
**3**	82.3	35.9	17.4	12.9
**4**	92.4	88.2	53.3	24.5
**6**	88.7	48.6	21.2	17.9
**7**	91.6	89.0	54.2	22.6
**8**	18.7	4.0	3. 6	-
**9**	90.9	58.0	31.8	15.5
**10**	87.0	64.6	30.1	12.4
**12**	94.0	53.8	24.7	20.0
**Trolox**	92.0	90.0	38.0	22.0

^a^ After 30 min of incubation. All determinations were performed at least in triplicate, and standard deviation is always within ±10% of the mean value. -: no interaction.

**Table 3:** Effect of compounds **1**–**12**, ibuprofen and ketoprofen on carrageenan-induced rat paw edema ^a^.

Compound	% Edema Reduction
**1**	43 **
**2**	44 **
**3**	27 *
**4**	37 *
**5**	47 *
**6**	44 *
**7**	45 **
**8**	48 **
**9**	52 ***
**10**	42 *
**11**	67 ***
**12**	59 ***
**Ibuprofen**	36 *
**Ketoprofen**	47 **

^a^ The effect on edema is expressed as percent of inhibition of edema in comparison to controls. All compounds were administered i.p. at a dose of 0.15 mmol/kg of body weight. Each value represents the mean obtained from 6 animals. Significant difference from control: * *p* < 0.02, ** *p* < 0.005, *** *p* < 0.001 (Student’s *t* test).

**Table 4:** Effect of compounds **1**–**12**, ibuprofen, ketoprofen, BHT and NDGA on lipoxygenase ^a^.

Compound	IC_50_ (μM) or %/100 μM
**1**	30%
**2**	15%
**3**	20%
**4**	15%
**5**	200
**6**	156
**7**	-
**8**	13
**9**	80
**10**	23%
**11**	86
**12**	90
**Ibuprofen**	200
**Ketoprofen**	220
**BHT**	192
**NDGA**	1.3

^a^ After 7 min of incubation; ΒHΤ: butylated hydroxytoluene; NDGA: nordihydroguaiaretic acid; -: inactive. All determinations were performed at least in triplicate, and standard deviation is always within ±10% of the mean value.

**Table 5:** Effect of compounds **4**, **5**, **9**, **11**, **12** and simvastatin on Triton WR1339 (tyloxapol)-induced hyperlipidemia.

Compound	% Reduction			
	Dose (i.p.) (μmol/kg)	TC ^a^	TG ^b^	LDL-C ^c^
**4**	150	46.2 **	53.4 **	60.2 *
**5**	150	52.3 **	62.0 **	66.0 **
**9**	150	61.2 **	52.1 **	70.1 *
**11**	150	57.6 **	44.5 *	51.2 **
**12**	150	53.4 **	33.2 *	56.2 *
**Simvastatin**	150	73.0 **	-	70.0 **

^a^ TC: total cholesterol; ^b^ TG: triglycerides; ^c^ LDL-C: LDL cholesterol; Tyloxapol: 200 mg/kg, i.p.; significant difference from hyperlipidemic control group: * *p* < 0.05, ** *p* < 0.01 (Student’s *t* test).

## Data Availability

The data presented in this study are available on request from the corresponding author.

## Sample Availability

Samples of the compounds are not available from the authors.
